# A new species of *Palpita* (Crambidae, Spilomelinae) from the coastal plains of southeastern United States

**DOI:** 10.3897/zookeys.264.4363

**Published:** 2013-02-06

**Authors:** J. Bolling Sullivan, M. Alma Solis

**Affiliations:** 1 200 Craven Street, Beaufort, North Carolina 28516 USA; 2 Systematic Entomology Laboratory, PSI, Agricultural Research Service, U. S. Department of Agriculture, c/o National Museum of Natural History, Smithsonian Institution, P. O. Box 37012, MRC 168, Washington, DC 20013 USA

**Keywords:** Taxonomy, *Palpita*, Spilomelinae, coastal plain, North Carolina, Florida, Alabama

## Abstract

A new species of *Palpita* Hübner (Crambidae, Spilomelinae), *Palpita maritima*, **sp. n.,** is described from maritime forests of the coastal plains of southeastern United States.

## Introduction

The genus *Palpita* Hübner is distributed worldwide. In the United States it consists of 11 recognized species (Munroe 1952, 1959, 1983). In North Carolina 6 species have been recorded, but only *Palpita magniferalis* (Walker) is found with some frequency. The other five species are very infrequently encountered. An exception, however, occurs in April along the coast in maritime forest habitats where numerous individuals of a *Palpita* species were collected by the first author. Genitalic morphology and molecular COI barcode analyses indicate these specimens represent a species new to science that is described below.

## Material and methods

Specimens are deposited in the following collections: JBS (J. Bolling Sullivan, North Carolina, USA) and USNM (National Museum of Natural History, Washington, District of Columbia, USA).

Photographic methods used are described in Sullivan and Adams (2009). Procedures for dissecting and preparing genitalia follow Lafontaine (2004). DNA sequencing of the barcode fragment of the COI gene was conducted at the Canadian Center for DNA Barcoding in Guelph, Ontario. Barcode sequences (LNCC1234-11, LNCC1235-11) were compared by Nearest Neighbor Analyses as implemented on the Barcode of Life Data systems website (Ratnasingham and Hebert 2007).

## Systematics

### Palpita maritima

Sullivan & Solis sp. n.

urn:lsid:zoobank.org:act:16C4A455-EB87-4BAD-913F-9358B54271FB

http://species-id.net/wiki/Palpita_maritima

Figs 1–3

#### Type material.

**Holotype** male: **USA: North Carolina,** DCM Properties, Bald Head Island, Brunswick County (33.853; -79.9752), 31 March 1994, J. Bolling Sullivan, Richard Broadwell, Brad Smith (USNM). **Paratypes:** 13 males, 2 females: same data as type. 10 m, 31 March 1994; 3 males, 2 females: 13 April 1994 (USNM).

#### Additional material examined.

**North Carolina**, Carteret Co. Roosevelt Natural Area, Bogue Bank, 10-IV–2008. **Florida,** Putnam Co., Welaka For. Cons. Area, 17–21-III-1986, J.B. Heppner, Welaka Site 5, slashpine palmetto flatwoods. Liberty Co., Torreya State Park, 30-III-1988, H.D. Baggett. **Alabama,** Baldwin Co., Blakely State Park (30.749; -87.0142), 25–29-VII-2011, J. Bolling Sullivan.

#### Diagnosis.

The male genital characters, especially the shield-like juxta with two posterior pointed projections and the medial ribbon-like sclerotization across the valva, are diagnostic. External maculation, while fairly distinct within the genus, will not always distinguish this species readily from some forms of *Palpita arsaltealis* (Walker) and *Palpita freemanalis* Munroe that are also known from eastern U.S. coastal areas.

#### Description.

Male. *Head* Labial palpi brown scaled above, white scaled below, scaling on inner surface lighter brown. Haustellum white scaled. Frons brown scaled with darker, chocolate-colored patches laterally. Vertex with central white scaling and lateral brown scaling. Maxillary palpi developed, forming mesially directed tufts at occipital angle. Eyes large with well-developed corona. Ocellus present. Antenna brown scaled dorsally to tip and tan ventrally with scape brown, pedicel brown with white shining scales at base; fasciculate with tiny ventral setae. *Thorax and abdomen* Thorax with fuscous scaling. First two abdominal segments with white scaling dorsally, fuscous and chocolate scaling laterally. Remaining segments chocolate colored dorsally with scattered fuscous scales covering most of segment and a row of white scales distally giving abdomen a ringed appearance. Terminal segment largely fuscous. Underside of abdomen white with scattered fuscous scales. Abdomen extends 1/3 length beyond wing margins. *Wings* (wing length=12 mm, n=20); span (wing tip to wing tip=29 mm). Forewing with apex slightly rounded. Ground color brown, a mixture of chocolate and fuscous scales. Orbicular and reniform spots well marked. Wing pattern varies depending on condition of wear. Some individuals with well-marked chocolate-colored areas. Hindwing fuscous, less patterned than forewing. Underside of wings white, less patterned, but orbicular and reniform spots visible. *Legs* Forelegs with alternating brown and white-scaled regions. Middle legs brown dorsally, white ventrally. Hind legs white. A single pair of spurs on mid tibia, two pair on hind tibia with smaller, distal spurs brown, proximal spurs white. Some individuals with all spurs white. Female similar to male, scaling on leg spurs usually white with scattered brown scales. *Male Genitalia* Tegumen posterodorsally square. Uncus elongate, narrow in middle, wider at both ends. Distal end smoothly rounded with a pad of dorsal setae. Valva broadly rounded with moderately-dense hair patch at apex. Costa sclerotized, slightly narrower than width of apex of uncus and widening slightly at 2/3 from base. Sacculus heavily sclerotized, half width of valva at base, narrowing distally, not extending to costal apex, with three dorsal (toward costa) projections. Most distal projection tapers at tip and extends thumb-like medially, extends toward costa more than ½ distance but shape variable among individuals and between left and right valves. Most basal projection about half distance along length of valva, broadly rounded, extends less than ½ way to costa. Between these two projections, a less-sclerotized thin, ribbon-like projection extends across valva and bends toward costa without touching costa. Medial area of valva unsclerotized, with broadly-spaced setae. Vinculum 2–3× broader than tegumen, parategumen sclerites (=a pair of lateral sclerites located in basal region of tegumen, usually with long pencils or brushes of scales (=coremata) (Clavijo 1990)) present. Saccus pointed, curving dorsad. Juxta large, shield-like with distinct sub-basal invaginations. Posterior juxtal tip broad with two distinct pointed projections. Phallus weakly sclerotized; apex without projection or ornamentation on shaft. Ductus seminalis subbasal. Vesica sac-like, slightly wider than phallus with a dorsal basal sclerotization often looking like a fishing hook. A larger and more distal ventral patch of deciduous cornuti present (in some specimens only sockets remain on a sclerotized patch). *Female Genitalia* Eighth segment trapezoidal, 5× as wide as high with numerous setae arranged in 4 somewhat irregular rows. Anterior apophyses about 3× as long as posterior apophyses. Anterior and posterior apophyses narrow, rounded at tips. Seventh segment ovoid, lightly sclerotized. Ostium bursae broad, about 4/5 width of segment, flat, and extending dorsally with short sclerotized spines at lateral edges. Ductus bursae short, posteriorly highly sclerotized, anteriorly lightly sclerotized, slightly sclerotized at junction with corpus bursae. Corpus bursae lightly sclerotized anterior to ductus bursae. Two ventrally located, elongated, horn-shaped signa anterior to base of corpus bursae. Corpus bursae membranous, elongate, about half length of genital apparatus.

**Figures 1–4.**
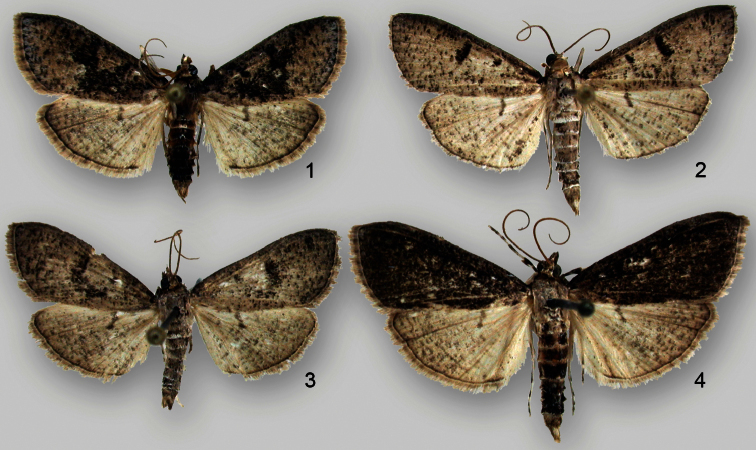
*Palpita maritima*, adult males from Bald Head Island, Brunswick County, North Carolina showing variation in maculation **1–3** paratypes **4** holotype.

**Figure 5–6.**
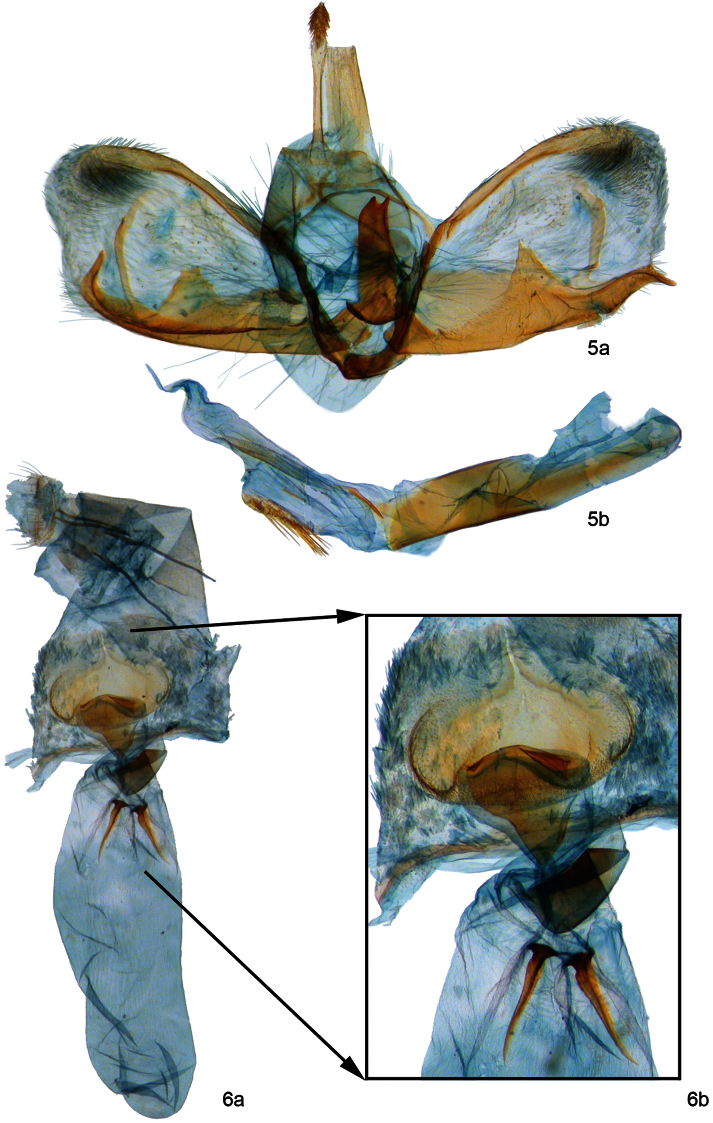
**5** *Palpita maritima*. A. male genitalia showing parategumen sclerites (= coremata). B. phallus with everted vesica **6** *Palpita maritima* A. female genitalia. B. ostium bursae region.

#### Biology and distribution.

The species has been found frequently in North Carolina from Carteret County south to Brunswick County in late March and early April in coastal maritime forests. Additional captures have been made in the same habitat in June, July and August, but very few individuals represent later broods. This species has a much broader range along the southeastern coastline, but has been confused with other species and thus has remained unknown. For example, from Florida two specimens from Archbold Preserve, Highlands County, have barcodes that match *Palpita maritima* and two specimens from Liberty and Putnam counties morphologically match *Palpita maritima*. A male from Baldwin Co., Alabama, has been dissected and matches the North Carolina holotype. These habitats are dominated by live and laurel oaks, loblolly pines, yaupon holly and *Smilax* species. The larval foodplants of *Palpita maritima* have not been discovered, but other species of *Palpita*, including *Palpita gracilialis* (Hulst), *Palpita kimballi* Munroe, *Palpita magniferalis* and *Palpita quadristigmalis* (Guenée), use species of Oleaceae as foodplants (Robinson et al. 2002). *Osmanthus americana* (L.), Wild Olive, is distributed in the maritime forests of the outer Coastal Plain and is the likely food plant.

#### Remarks.

Nearest neighbor joining barcode trees place *Palpita maritima* in a group that includes the *Palpita arsaltealis* (Walker) complex and *Palpita illibalis* (Hübner). *Palpita maritima* was compared to multiple genitalic preparations at the USNM of *Palpita arsaltealis* and *Palpita illibalis*. Males of *Palpita arsaltealis* (see fig. 3, Munroe 1952) and *Palpita illibalis* (see fig. 4, Munroe 1952)have a shield-like juxta, but lack pointed projections posteriorly that occur in *Palpita maritima* males (Fig. 5). The valvae of *Palpita arsaltealis* and *Palpita illibalis* are not as complex as *Palpita maritima*. *Palpita arsaltealis* and *Palpita illibalis* have two longer, pointed saccular projections. *Palpita maritima* has three saccular projections, one distal, short, and pointed, and another proximal, short, and broad. *Palpita arsaltealis* and *Palpita illibalis* both lack the medially-located thin, ribbon-like sclerotization extending almost the width of the valva toward the costa that occurs in *Palpita maritima*. *Palpita maritima* females have lightly and incompletely sclerotized ductus bursae and the signa are ventrally located, unlike *Palpita arsaltealis* (see fig. 10, Munroe 1952) and *Palpita illibalis* (see fig. 11, Munroe 1952) females that have lightly but more completely sclerotized ductus bursae, and the signa are located almost laterally in the corpus bursae. *Palpita maritima* females (Fig. 6) have anterior apophyses three times as long as posterior apophyses, whereas *Palpita arsaltealis* and *Palpita illibalis* females have anterior apophyses that are two times as long as the posterior apophyses.

#### Etymology.

The name refers to the habitat type, coastal maritime forest, where the species is most abundant in the spring.

## Supplementary Material

XML Treatment for Palpita maritima
